# Evaluation of a Brief Sleep Intervention Designed to Improve the Sleep, Mood, and Cognitive Performance of Esports Athletes

**DOI:** 10.3390/ijerph19074146

**Published:** 2022-03-31

**Authors:** Daniel Bonnar, Sangha Lee, Brandy M. Roane, Daniel J. Blum, Michal Kahn, Eunhee Jang, Ian C. Dunican, Michael Gradisar, Sooyeon Suh

**Affiliations:** 1 College of Education, Psychology, and Social Work, Flinders University, Adelaide, SA 5042, Australia; daniel.bonnar@flinders.edu.au (D.B.); michal.kahn@flinders.edu.au (M.K.); 2 Department of Psychiatry, Ajou University School of Medicine, Suwon 16499, Korea; xrpsychology@gmail.com; 3 Department of Pharmacology and Neurosciences, UNT Health Science Centre, University of North Texas, Fort Worth, TX 76107, USA; brandy.roane@unthsc.edu; 4 Department of Arts and Sciences, New York University Shanghai, Shanghai 200122, China; drblum@sleepwise.io; 5 Department of Psychology, Sungshin Women’s University, Seoul 02844, Korea; eunhee2914@gmail.com; 6 Centre for Sleep Science, School of Human Sciences, The University of Western Australia, Perth, WA 6009, Australia; iandunican@sleep4performance.com.au; 7 WINK Sleep Pty Ltd., Adelaide, SA, Australia; grad0011.mg@gmail.com

**Keywords:** esports, sleep, performance, intervention, mood, cognitive

## Abstract

This study evaluated a brief sleep intervention designed to improve the sleep, mood, and cognitive performance of professional electronic sports (esports) athletes from three major esports regions (i.e., Asia, North America, and Oceania). Fifty-six esports athletes from South Korea (*N* = 34), the United States (*N* = 7), and Australia (*N* = 15) completed the study. Participants completed an initial 2-week pre-intervention phase to establish a baseline, followed by a 2-week intervention phase that involved a group sleep education class, 1:1 session with a trained clinical psychologist, and daily biofeedback. A wrist activity monitor and daily sleep diary were used to monitor sleep during both phases, while at pre- and post-intervention, participants completed a battery of sleep and mood questionnaires and underwent cognitive performance testing. Sleep knowledge increased from pre- to post-intervention (d = 0.83 [95% CI −1.21, −0.43], *p* =< 0.001), while there were modest improvements in sleep diary estimates (i.e., sleep onset latency (*M*_diff_ = −2.9 min, *p* = 0.02), sleep onset time (*M*_diff_ = −12 min, *p* = 0.03), and sleep efficiency (*M*_diff_ = 1.1%, *p* = 0.004)) and wrist activity monitor estimates (i.e., sleep onset time (*M*_diff_ = −18 min, *p* = 0.01)). Insomnia severity scores decreased significantly (d = 0.47 [95% CI 0.08, 0.84], *p* = 0.001), while sleepiness scores increased but not meaningfully (d = 0.23 [95% CI −0.61, 0.14], *p* = 0.025). However, there was no significant change in mood (i.e., depression and anxiety) or cognitive performance scores (i.e., mean reaction time or lapses). Sleep interventions for esports athletes require further investigation. Future research should examine whether a stepped-care model, whereby increasing therapeutic input is provided as needed, can optimize sleep, mood, and cognitive performance outcomes.

## 1. Introduction

Electronic sports (esports) are a form of organized video game competition that have become increasingly popular and professionalized over the last decade [1]. The global esports industry is predicted to generate USD 1.084 billion in revenue and reach a worldwide audience of 474 million in 2021 [2]. In response to this growth, researchers have begun to investigate factors that influence the performance and well-being of professional competitors—known as esports athletes. Within this budding field of academic enquiry, the function of sleep has emerged as a particular area of interest, due to its central role in supporting human health (physical and mental) and performance [3,4]. However, the unique characteristics and conditions experienced by esports athletes, including, but not limited to, long training hours (mean = 9.2 h per day) [5], performance stress [6], and a tendency toward a late chronotype (i.e., a delayed sleep pattern) [5], may increase the risk of sub-optimal sleep outcomes. 

A small but growing body of evidence on the sleep patterns of esports athletes has been documented. Studies [5,6,7] comprising participants from South Korea, Australia, Brazil, and the United States (US) have found that esports athletes have significantly delayed sleep timing (e.g., median = 3:43–11:24 a.m. [5]). Their total sleep time (TST) is low, ranging from 6.5 to 7.2 h per night, with sleep diary estimates being greater than actigraphy estimates. Sleep onset latency (SOL) is typically adequate (i.e., <30 min [8]) when measured with sleep diary and wrist actigraphy. However, while sleep diary estimates of wake after sleep onset (WASO) are within normal limits (i.e., <20 min [8]), actigraphy estimates are excessive, which may reflect the discrepancy between these measures observed in the sleep literature [9]. Collectively, these findings suggest that some esports athletes have delayed sleep timing and are sleep-restricted, which aligns with subjective estimates of high daytime sleepiness [5,6]. 

There are two important implications for esports athletes who obtain poor sleep. First, sleep restriction has a well-known deleterious effect on cognitive performance, including slower reaction times, increased memory lapses, decreased vigilance and attention, and impaired executive functioning [10,11,12]. Importantly, these aforementioned cognitive abilities (amongst others) have been proposed to play a role in esports, in which games are often fast-paced, require complex decision making and adaptation, and can be played for varying periods of time (i.e., 5–45 min) over successive matches during multi-day tournaments [1]. Second, some research has shown a relationship between esports and poor mental health outcomes [13]. Sleep restriction contributes to disturbed mood states (e.g., depression and anxiety) [14,15], with preliminary evidence showing a possible link between sleep and depressive symptoms in esports athletes [5,6]. Thus, esports athletes’ performance and mental health could be compromised due to poor sleep. 

Sub-optimal sleep experienced by esports athletes and the potential consequences for performance and mood suggest that sleep interventions are warranted. We have previously proposed that cognitive behavior therapy for insomnia (CBT-I), a multi-component treatment package, could be adapted to meet the needs of esports athletes [16]. CBT-I treatment components include education to improve sleep knowledge, cognitive techniques to reduce nocturnal arousal levels, behavioral strategies to establish consistent sleep scheduling, and motivational tasks to enhance treatment compliance [16], which are usually delivered over 4–8 weekly sessions. However, the demands of training and the competition schedules to which esports athletes are required to adhere [5] suggest that a brief low-intensity (i.e., small-dose) sleep intervention, which has shown to be effective in traditional athlete populations [3], may be more feasible and realistic for teams to accommodate. 

Moreover, since esports athletes are young (e.g., mean = 20 yrs [5]) and therefore inclined to be high technology users, biofeedback may be an engaging adjunctive intervention component. In a sleep context, biofeedback typically refers to a wrist-worn activity monitor being paired with a smartphone application to relay sleep (and sometimes subsequent cognitive performance) data back to an individual following a period of sleep [3]. Biofeedback has shown mixed results in the treatment of sleep disorders such as insomnia [17]. However, it has been recommended, using an appropriately validated wrist activity monitor, for traditional athletes as a non-intrusive means of increasing sleep awareness [3]. The benefit of increased sleep awareness for esports athletes participating in a sleep intervention is that it would enable the active monitoring of progress while simultaneously making sleep-related behavioral changes.

The present study aimed to evaluate a brief low-intensity sleep intervention designed to improve the sleep, mood, and cognitive performance of esports athletes from three major esports regions (i.e., Asia, North America, and Oceania), using a single-arm open-label multi-center interventional study design. To the best of the authors’ knowledge, this is the first time a sleep intervention has ever been trialed with esports athletes in any context. We predicted that participants would obtain short-term improvements in sleep (e.g., TST), mood (i.e., depression and anxiety symptoms), and performance (i.e., mean reaction time and lapses) at post-intervention relative to pre-intervention.

## 2. Materials and Methods

### 2.1. Participants

Participants were South Korean, US, and Australian esports athletes. Top esports organizations (i.e., organizations that had teams in tier 1 and 2 leagues) in each country were contacted via email regarding the study. When an organization indicated interest by responding, potential participants were debriefed on what would be involved in the study, after which they provided informed written consent. Participant enrollment and study participation were completed in cohorts (i.e., team-based). The countries were selected because they are part of official esports regions (i.e., Asia, North America, and Oceania), and these are where the authors resided. Thirty-Four South Korean esports athletes have previously had their pre-intervention subjective sleep estimates reported [6], while a total of seventeen esports athletes from Australia, South Korea, and the US have previously had their pre-intervention objective sleep estimates reported [5]. Ethics approval was obtained in all three countries from relevant ethics boards, including the Flinders University Social and Behavioral Research Ethics Committee (project number: 8408), the Sungshin Women’s University Review Board (project number: SSWUIRB-2019-030), and the North Texas Regional Institutional Review Board (project number: 2019-165). Participants were eligible for inclusion in the study if they competed on a professional team as part of an official esports league. No exclusion criteria (e.g., sleep medication use) were used to increase the generalizability of the sample. 

### 2.2. Measures

#### 2.2.1. Demographic/General Information

A self-report questionnaire was used to measure demographic and general information, including self-reported anthropometric data (i.e., height (cm) and weight (kgs)) used to calculate BMI values, esports background (i.e., career length and training hours (i.e., total number of hours, initial start and final stop times for the day)), sleep history (i.e., sleep medication use), self-reported caffeine use in milligrams (mg) per day, and self-reported sleep disturbance the night before a competition (in the last year). 

#### 2.2.2. Sleep Measures

##### Wrist Activity Monitor

Wrist activity monitors (i.e., Readiband V5 Fatigue Science Inc., Vancouver, BC, Canada) were used to obtain an objective measure of esports athletes’ sleep. Analyses of the wrist activity monitor yield raw acceleration signals with a proprietary algorithm to generate sleep/wake data. Evidence suggests that it adequately compares to both gold-standard in-laboratory polysomnography and several other popular wrist activity monitors [18]. Sleep data derived from the wrist activity monitor included SOL, sleep onset time, WASO, sleep efficiency (i.e., TST/time in bed × 100; time in bed was calculated from the time participants’ attempted sleep to their wake-up time), TST, time in bed, and wake-up time. Accelerometer data were also used to establish sleep timing (i.e., sleep onset time, wake-up time [19]). 

##### Sleep Diary

The American Academy of Sleep Medicine recommends that sleep diaries be used in conjunction with wrist activity monitors [20]. Hence, a daily online sleep diary based on the Consensus Sleep Diary developed by Carney et al. [21] was administered upon waking at the end of each major sleep period. The questions were related to bedtime, lights-out time, sleep onset time, WASO, and wake-up time. These data were also used to calculate additional sleep variables, including SOL, TST, sleep efficiency (i.e., TST/time in bed × 100; time in bed was calculated from the time participants attempted sleep to their wake-up time), and sleep timing (i.e., sleep onset time, wake-up time).

##### Insomnia Severity Index (ISI)

The ISI [22] is a 7-item self-report instrument used to assess the presence and severity of insomnia in participants. Items (e.g., “*How worried/distressed are you about your current sleep problem*”) are rated on a 5-point Likert scale from 0 (e.g., “*not at all*”) to 4 (e.g., “*very much*”) and summed to generate a total score. Higher total scores (range 0–28) indicate greater insomnia severity. The ISI has sound internal consistency (in the present study, α = 0.75 across time), test–retest reliability, and construct validity, while a cut-off score of 10 had 86.1% sensitivity and 87.7% specificity for detecting insomnia cases in community samples [23]. A Korean version of the ISI was used [24].

##### Pediatric Daytime Sleepiness Scale (PDSS)

The PDSS [25] is an 8-item self-report instrument used to assess levels of daytime sleepiness in participants. Wording of items 1 and 2 were altered to increase relevance for esports athletes, as the original items were school-related, and no participants were attending school. Items (e.g., “*How often do you fall back to sleep after being woken in the morning?*”) are rated on a 5-point Likert scale from 0 (“*never*”) to 4 (“*always*”). Item 3 is reverse-scored. Item scores are summed to generate a total score (range 0–32,) with higher total scores indicating greater daytime sleepiness. The PDSS has good internal consistency (in the present study, α = 0.78 across time) and is correlated with reduced sleep duration [25]. The PDSS was chosen over other popular sleepiness scales (e.g., Epworth Sleepiness Scale (ESS)) due to its items showing greater face validity (i.e., questions aimed at morning sleepiness vs. situations in the ESS such as being in a car stopped in traffic). It has been used in samples with participants up to age 24 years [26] and with esports athletes [5]. A Korean version of the PDSS was used [27].

##### Sleep Knowledge

Sleep and performance knowledge was measured using a quiz adapted from Bonnar et al. [28] (see Supplementary Material). Participants answered “*true*”, “*false*”, or “*don’t know*” to 16 items relating to information about sleep and performance (e.g., “*Most young adults need between 7–9 h sleep per night”*). Correct answers were scored 1 and incorrect and “*don’t know*” responses as 0. Individual item scores were summed to generate a total score out of 16, with higher total scores indicating greater knowledge. In the present study, the Kuder–Richardson Formula 20 coefficient was adequate (α = 0.69 across time). This measure was translated into Korean and back-translated by a bi-lingual member of the research team.

#### 2.2.3. Mood Measures

##### Centre for Epidemiological Studies Depression (CES-D)

The CES-D [29] is a 20-item self-report instrument used to assess depressive symptomatology. Items (e.g., “*I was happy*”) are rated on a 4-point Likert scale from 0 (“*rarely or none of the time*”) to 3 (“*most or all of the time*”). Individual item scores were summed to generate a total score ranging 0–60, with higher total scores indicating greater levels of depressive symptoms. A cut-off score of ≥16 was used as an indicator of depression. The CES-D has adequate internal consistency (in the present study, α = 0.76 across time), test–retest reliability, and construct validity [29,30]. A Korean version of the CES-D was used [31].

##### State-Trait Anxiety Inventory (STAI-Y)

The STAI-Y [32] consists of 2 anxiety scales (i.e., trait and state), with the 20-item self-report state scale used to assess state anxiety in the current study. Items (e.g., “*I am tense*”) are rated on a 4-point Likert scale from 1 (“*not at all*”) to 4 (“*very much so*”). Individual item scores were summed to generate a total score ranging 20–80, with higher total scores indicating greater levels of state anxiety. The STAI-Y has adequate psychometric properties, with sound internal consistency (in the present study, α = 0.92 across time), test–retest reliability, and construct validity [33,34]. A Korean version of the STAI-Y was used [35].

#### 2.2.4. Cognitive Performance Measure

##### Psychomotor Vigilance Task (PVT-5)

The PVT-5 is a measure of vigilant attention [36] that was delivered on an iPad using Joggle Research software V2.7 (Seattle, WA, USA). Participants were presented with a screen with a red rectangle in the center, in which a visual stimulus (i.e., yellow counter) appeared randomly every 2–10 s over a 5 min trial period (participants completed 49–51 responses depending on their reaction time). The participant was required to respond to the appearance of the on-screen counter by touching the screen as quickly as possible. Once the screen had been touched, the counter was stopped, and the reaction time (in ms) displayed for 1 s. False starts or a reaction time <100 ms were labeled “errors”. Based on previous research (e.g., [37]), lapses were defined as a reaction time ≥500 ms. The primary outcomes of interest from the PVT-5 were mean reaction time and number of lapses per 5 min trial period. The PVT-5 is considered an acceptable substitute for the gold-standard PVT-10 based on evidence showing similar performance between the two tests [38], which has also been established using Joggle Research software [37]. 

### 2.3. Procedure

**Figure 1:**
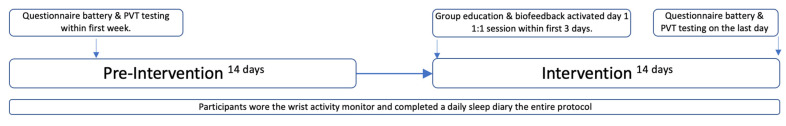
Study procedure flow from the pre-intervention to intervention period.

The study protocol took place during a regular competitive season in each team’s respective league. Hence, participants were actively training and competing at the time of the study, thereby maximizing ecological validity. Data were collected between July 2019 and March 2020 (prior to COVID-19 impacting each country). Participants were assigned an anonymized study ID for all data collection points. Figure 1 outlines the study procedure.

#### 2.3.1. Pre-Intervention Period

Participants completed 14 days of pre-intervention data collection, except for one US team (5 participants) that completed 7 days due to a conflict with a major tournament. Participants were instructed to maintain their typical sleep–wake behavior during this time. They continuously wore the wrist activity monitor on their non-dominant wrist (unless impractical to do so, such as when showering) and completed an online daily sleep diary via a weblink. Sleep diary entries were monitored by a member of the research team, and if participants had not completed their sleep diary by late afternoon (afternoon was selected due to some participants having very delayed circadian timing), they were contacted and reminded to do so. Participants also completed a battery of questionnaires (i.e., ISI, PDSS, CES-D, STAI-Y, and sleep knowledge quiz, via paper or online with Qualtrics) and underwent cognitive testing individually with a member of the research team within the first week (testing for each team occurred within a 12–9 p.m. window) to establish baseline scores for the pre-intervention period. 

#### 2.3.2. Intervention Period

Following the pre-intervention period, participants completed a 14-day intervention period. On Day 1 of the intervention period, participants completed a group sleep education class. The class included a presentation that outlined information on the importance of sleep for health and performance in esports, sleep physiology, sleep hygiene, sleep myths (e.g., waking during the night is abnormal), characterization of adequate sleep, and a brief introduction to brief behavioral therapy for insomnia. The class was 40 min long and conducted by a clinical psychologist specializing in behavioral sleep medicine (i.e., authors D.B., S.S., S.L., B.R., or D.J.B.; experience ranged 5–15 years).

At the end of the group presentation, participants downloaded a phone application (Fatigue Science) and paired it with their wrist activity monitor. The app enabled participants to access biofeedback on their phone (i.e., their sleep data). The Readiband has an additional function that allows captured sleep data to be analyzed to produce a daily cognitive performance score ranging 0–100, which is also relayed to the user via the app [39]. Higher scores indicate faster and more accurate reaction times [39]. For further information about the cognitive performance function’s use and validation, see Hursh et al. [39], Hursh et al. [40], and Van Dongen [41]. Participants were encouraged to check the app daily to track their progress. Use of the app was not monitored. 

Between days 1–3 of the intervention period, each participant completed a 30 min 1:1 session with the same clinical psychologist who conducted the group education. Sessions occurred over 3 days to accommodate busy team training schedules. During the 1:1 session, participants were provided with a brief report that outlined a summary of their sleep and cognitive performance data from the wrist monitor obtained during the baseline period. Data included sleep variables (e.g., sleep onset time, SOL, WASO, TST, wake-up time) and average cognitive performance score for each day. The clinical psychologist described four recommendations adapted from brief behavioral therapy for insomnia [42]. These included the following: (1) reduce time in bed if sleep quality is poor; (2) keep a consistent wake-up time; (3) do not go to bed unless sleepy; and (4) do not stay in bed unless you are sleeping [42]. A relaxation exercise (i.e., diaphragmatic breathing for 2–3 min) was practiced to help esports athletes reduce pre-sleep arousal levels if needed [43]. At the end of the session, three motivational interviewing questions based on the themes of desire, ability, and commitment (e.g., “*how confident are you about improving your sleep?*”) were used to enhance motivation and treatment compliance [44]. Finally, a sleep and performance manual that summarized the content covered in the group education and individual 1:1 session was provided as a reference. Participants were encouraged to read the manual, and use the information gained in the group education and 1:1 session to try to improve their sleep until the conclusion of the intervention period. 

On the last day of the intervention period, participants returned their wrist activity monitor, completed a battery of questionnaires (i.e., ISI, PDSS, CES-D, STAI-Y, and sleep knowledge quiz, via paper or online with Qualtrics) and underwent cognitive testing. Cognitive testing for each team occurred within 2 h of their pre-intervention testing time. 

### 2.4. Data Analysis

Descriptive statistics, including frequencies, means, and standard deviations, were used to summarize sample characteristics. Data for all variables were inspected for missing values and outliers prior to commencing data analysis. Missing data for sleep diary variables ranged 2.7–2.8%, while missing data for wrist activity monitor variables ranged 13.9–14.5%. Unreliable data due to technical issues were removed. Linear mixed-model regressions were used to test the main effect of time on all primary outcome measures, with SPSS v.26.0 (Armonk, NY, USA) used for questionnaires and PVT, and R v3.6.3 (R Foundation for Statistical Computing, Vienna, Austria) and RStudio v1.2.5033 (Boston, MA, USA) used for sleep diary and wrist activity monitor data, due to their nested structure (multiple nights per assessment period). Linear mixed-model regressions accounted for missing data using maximum likelihood estimation. All linear mixed-model regressions adjusted for participant age, gender, body mass index (BMI), training hours, and country. A significance level of *p* ≤ 0.05 was used. Effect sizes (i.e., Cohen’s d for questionnaire scores and PVT number of lapses per trial, and mean difference (*M*_diff_) for sleep variables and PVT mean reaction time) were calculated for primary outcome measures. 

## 3. Results

### 3.1. Sample Characteristics

Participants were South Korean (*N* = 34), US (*N* = 7), and Australian (*N* = 15) esports athletes (for sample characteristics, see Table 1). The response rate was 95% (56/59). A total of 57 participants initially commenced the study. One participant from Australia was excluded (i.e., left their team during the pre-intervention period). Thus, a total of 56 participants were included in the final analysis. Most participants were male (*N* = 54, 96.4%), with mean age of 20.9 years ± 2.4 (range = 14–27), and the mean BMI was 23.8 ± 5.3 (range = 16.1–37). There was no significant difference in age or BMI between countries. Participants’ mean career as a professional esports athlete was 2.6 years ± 1.6 (range = 0–9). Participants competed in either first-person shooter games (*N* = 24), massive online battle arena games (*N* = 22), battle arena games (*N* = 6), sport games (*N* = 3), or fighting games (*N* = 1). Korean participants had significantly longer training hours than Australian (*M*_diff_ = 6.91, standard error (SE) = 0.63, *p* < 0.001) and US participants (*M*_diff_ = 5.78, SE = 0.84, *p* < 0.001). The difference between Australian and US training hours was non-significant (*M*_diff_ = 1.13, SE = 0.93, *p* = 0.23). 

With respect to training timing, on average, Korean participants trained between 1 p.m. and 2 a.m., Australian participants trained between 4 and 11 p.m., and US participants trained between 12 and 6 p.m. Regarding sleep aids, only one participant from Australia used melatonin. 

### 3.2. Sleep Knowledge

There was a significant main effect of time for the sleep knowledge quiz (d = 0.83 95% CI [−1.21, −0.43], *p* ≤ 0.001). From pre- to post-intervention, participants’ sleep knowledge improved by 14.3%. 

### 3.3. Sleep Diary

Daily sleep diary estimates are presented in Table 2. There was a significant main effect of time for SOL, sleep onset time, and sleep efficiency. From pre- to post-intervention, participants initiated sleep 2.9 min quicker on average, fell asleep 12 min earlier, and slept 1.1% more efficiently. There was no significant main effect for TST, WASO, or wake-up time. 

### 3.4. Wrist Activity Monitor

Wrist activity monitor estimates are presented in Table 3. There was a significant main effect of time for sleep onset time, whereby participants fell asleep 18 min earlier on average at post-intervention compared to baseline. The main effects for time were non-significant for TST, SOL, WASO, wake-up time, and sleep efficiency. 

### 3.5. Sleep, Mood, and PVT Scores

Sleep (i.e., ISI, PDSS), mood (i.e., STAI-Y, CES-D), and PVT (i.e., mean reaction time, lapses) scores are presented in Table 4. There was a significant main effect of time for the ISI and PDSS. From pre- to post-intervention, participants’ ISI scores decreased by 2.0 (i.e., participants reported fewer insomnia symptoms), and PDSS scores increased by 1.3 (i.e., participants reported higher sleepiness levels). There was no main effect of time for mood or PVT scores.

## 4. Discussion

An accumulating body of evidence suggests that esports athletes experience sub-optimal sleep, characterized by delayed sleep timing and restricted TSTs, with direct implications for mood and performance [5]. To the best of our knowledge, this study is the first to trial a brief low-intensity sleep intervention specifically designed to improve the sleep, mood, and performance of esports athletes over a short-term period. Overall, mixed results were observed. Based on self-report questionnaires, participants improved their sleep knowledge, reduced the severity of their insomnia symptoms, yet might have experienced greater sleepiness. In terms of sleep measures, sleep diary estimates showed improvements in SOL, an earlier sleep onset time, and improved sleep efficiency, while wrist activity monitor estimates corroborated the earlier sleep onset time. However, these modest improvements in sleep did not confer any mood (i.e., depression and anxiety) or cognitive performance (i.e., PVT) benefits. We now examine these findings in more detail and discuss considerations for future interventions.

### 4.1. Sleep Outcomes

Although there are no other sleep interventions in esports with which to compare our findings, similar brief low-intensity sleep interventions trialed in professional athlete samples (who are also sleep-restricted) offer a useful contrast. For example, Ryswyk et al. [45] provided an education session that outlined sleep hygiene information and strategies to increase TST and sleep quality, in addition to weekly sleep parameter feedback (e.g., SOL, WASO, TST, etc.) and a mid-intervention feedback session. Despite results showing subjective benefits for TST and sleep efficiency, there were no objective sleep benefits or change in PVT [45]. In another study, Driller et al. [46] provided a group education session and a 30 min 1:1 session that outlined personalized sleep hygiene recommendations. Results indicated improvements in subjective sleepiness and sleep quality and objective estimates of sleep efficiency, SOL, and sleep onset variance [46]. The findings from our study are more aligned with Ryswyk et al., in that there were mostly modest subjective short-term sleep benefits. Importantly, findings from Driller et al. combined with other recent evidence suggests that individualizing sleep interventions may produce more robust sleep benefits than a one-size-fits-all approach [3], and should thus also be investigated in esports. 

Notably, there was a small non-significant change (6–9 min) in TST at post-intervention. As part of the brief behavioral therapy for insomnia recommendations [42], esports athletes were encouraged to keep a consistent wake-up time, which our findings suggest they did. Hence, a further increase in TST was likely constrained by esports athletes’ difficulty in advancing sleep onset time more significantly than the 12–18 min (subjective and objective estimates) observed over the short timeframe. Given that there was a negligible change in SOL, this suggests that esports athletes did not dramatically shift the time they attempted sleep despite being mildly sleep-restricted. A previous qualitative study comprising Korean esports athletes found that late training times may increase arousal levels post-training, leading to bedtime procrastination characterized by technology use [6]. Recent evidence indicates that bedtime procrastination is related to delayed sleep timing, poor mood (anxiety and depression), and insomnia symptoms [47]. Hence, bedtime procrastination might have prevented esports athletes in our study from attempting sleep earlier when feeling sufficiently sleepy (as recommended in brief behavioral therapy for insomnia). Future research should quantify the level of bedtime procrastination in esports athletes (e.g., using the bedtime procrastination scale [48]) and investigate whether this does indeed interfere with their readiness to attempt sleep.

Consistent with the modest improvement in some subjective sleep estimates, there was a decrease in insomnia severity scores (i.e., ISI) to below the cut-off score of 10 at post-intervention. This decrease indicates that esports athletes perceived less sleep disturbance from pre- to post-intervention. In contrast, these improvements for subjective sleep were not reflected on our daytime sleepiness measure (i.e., PDSS), with participants experiencing possible increased sleepiness levels at post-intervention (i.e., results were significant but not meaningful). However, similar findings have been reported in previous research, whereby sleepiness scores initially increase at post-intervention, but then decrease at the 2-month follow-up [49]. Hence, future research should include a follow-up to monitor for lagged improvements in sleepiness levels.

Although not a primary focus of the present study, it should be noted that pre-intervention objective sleep estimates of participants are largely consistent with previously reported findings from our group involving a sub-set of esports athletes from Australia, South Korea, and the US [5] also involved in the present study. In both studies, participants from all countries were found to have significantly delayed sleep patterns and long WASO, leading to reduced TSTs of <7 h per night. There were some small discrepancies compared to our previous study, with SOL being slightly longer and sleep efficiency lower in the present study. However, the generally congruent findings between the initial smaller sub-set and our expanded sample increases confidence in the objective evidence obtained thus far regarding the sleep patterns of esports athletes in these countries.

### 4.2. Mood and Cognitive Performance Outcomes

Despite participants obtaining modest short-term sleep benefits, there was no subsequent change in mood or performance. Regarding mood, meta-analytic evidence indicates that non-pharmacological sleep interventions (e.g., individualized CBT-I) are effective in reducing symptoms of anxiety and depression [50,51]. Hence, in our study, either the benefits in sleep were too small to affect mood, or other factors (e.g., pressure to perform) unrelated to sleep might have been negatively impacting mood. Regardless, using sleep interventions to target mood in esports athletes remains a plausible mechanism to be tested further. Additionally, previous research investigating sleep interventions in adolescents with delayed sleep timing has demonstrated that an improvement in depression symptoms can lag [52]. In one study, despite improvements across multiple sleep parameters, there was no change in depression scores post-intervention, but there was at the 6-month follow-up [52]. Hence, future research could include a longer follow-up to monitor for delayed change in mood.

With respect to cognitive performance (i.e., PVT), the level of sleep restriction in our sample should first be highlighted. Esports athletes slept 6.9 (objective) and 7.4 (subjective) hrs per night on average. Van Dongen et al. found that <8 h per night led to cumulative, dose-dependent degradation in cognitive performance [11]. Thus, our study’s finding adds further weight to the existing evidence base [5,6,7] that some esports athletes are most likely competing while cognitively impaired.

In terms of the lack of PVT improvement, findings from the present study are consistent with previous sleep extension research showing that a large increase in TST (e.g., 1.3–1.7 h) can generate PVT improvement [53,54], while a smaller increase (e.g., 0.4 h) may not [55]. Although it is difficult to determine the exact TST change threshold at which PVT improves, the additional 6–9 min per night found in the present study appears insufficient. Furthermore, cognitive performance was limited to the PVT (i.e., mean reaction time and lapses) in the present study. There are a range of cognitive abilities that contribute to overall performance in esports that are also sleep-sensitive [1]. Thus, future sleep interventions should aim to include additional cognitive performance measures and possibly perceptual measures related to performance (e.g., motivation to perform).

### 4.3. Considerations for Future Sleep Interventions in Esports

The sleep intervention trialed in the present study was designed to be brief and low-intensity, so that it could be accommodated within the demanding training and competition schedule of esports athletes. Accordingly, the sleep education component was limited to a 40-min group education session, and the 1:1 session was 30 min long, while biofeedback allowed participants to maintain a passive awareness of their sleep. As mentioned earlier, similar brief low-intensity sleep interventions trialed in traditional athletes and consisting of group education and a 1:1 session have produced improvements in both subjective and objective sleep outcomes [45,46]. However, there may be a limit to what a brief low-intensity sleep intervention can achieve in an esports population. That is, although some esports athletes may experience benefits, others requiring greater therapeutic input may not.

A stepped-care model may be more effective in meeting the idiosyncratic sleep needs of esports athletes. In stepped-care models, an individual’s needs are assessed and matched against a hierarchy of interventions. This approach has become increasingly popular within the sleep health field in recent years [56] and has also been proposed for traditional athletes [3,57]. In esports, for example, all esports athletes could receive sleep education and then undergo assessment to determine which players require further therapeutic input and to what degree. Esports athletes identified as being “at risk” could participate in a brief low-intensity sleep intervention, while those found to be at “high risk” (e.g., diagnosed with insomnia) could receive more specialized therapy (e.g., individualized CBT-I). Importantly, a stepped-care model could feasibly fit within the busy lives of esports athletes. 

There are several other additions that could be included to improve the effectiveness of an esports sleep intervention. For example, existing organizational personnel, such as coaches and support staff (e.g., health and well-being officer, team psychologist, etc.), could be leveraged. Previous research has found that coaches and support staff in traditional sporting teams play a pivotal role in providing health- and performance-related advice to players, including sleep [58]. Hence, with appropriate training and guidance, esports coaches and support staff could participate in the delivery of a sleep intervention. Participation could include taking an active role in maintaining player engagement and accountability (e.g., checking in, encouragement, etc.), in a similar way to other aspects of training and team management. 

Another potential means of improving a sleep intervention’s effectiveness is to “gamify” educational and clinical content. Gamification could promote contextual learning, which refers to the concept that people learn best when new information being taught aligns with their existing frames of reference [59], as it enables the construction of meaning based on prior experience. Gamification has been used to increase physical activity [60] and could likewise be used to promote healthy sleep habits in a sleep intervention setting. For example, this could include modifying language to reflect esports nomenclature (e.g., suggesting players could obtain a sleep “buff”, which is a gaming term for a power increase), while using esports and gaming culture to shape intervention tools/resources (e.g., sleep variable leader boards) and the environment/delivery medium (e.g., esports discourse platforms).

The finding of improved sleep knowledge (14.5%) but only modest sleep benefits is consistent with several studies investigating group sleep interventions for young people. In these studies, increased sleep knowledge (ranging 8–13%) has not always translated into behavioral change, due to low motivation [61,62]. In the present study, motivational interviewing questions based on the themes of desire, ability, and commitment were included to enhance treatment compliance [44]. However, actual motivation levels were not measured. Hence, these motivational interviewing questions might have been insufficient to motivate participants to make greater changes to their sleep behavior. More relatable and engaging motivational tasks (e.g., playing a video game that simulates the impact of sleep restriction on performance) could enhance the intrinsic motivation to make sleep-related behavioral change. 

Finally, because sleep research in esports is in its infancy, there is still much to learn about the sleep experiences of esports athletes. As researchers gain a better understanding of the specific risk factors for sub-optimal sleep in esports, other more evidence-based intervention add-ons will become evident. For example, like traditional sports, it has been proposed that esports athletes who compete in different games may have varying risk profiles and associated sleep challenges [16]. Although available evidence is limited, preliminary findings suggest that training length and timing differs between games (and potentially region), which therefore displaces sleep patterns differentially [5]. Hence, a stronger focus on chronobiological strategies (e.g., light therapy, endogenous melatonin, etc.) could be more important for some esports athletes than others. 

### 4.4. Limitations

Although we have already discussed several limitations (e.g., the present study examined short-term effects with no long-term follow-up data) throughout the Discussion section, there are additional limitations worth noting. First, there was no control group. Thus, caution should be used when interpreting and generalizing our findings, with further research using more robust study designs being required before stronger conclusions can be drawn. Esports athletes are difficult to recruit given their demanding training and competition schedules. We found that esports organizations were strongly opposed to “burdening” players by having them participate as part of a control group that had no potential immediate benefit. Second, our sample primarily comprised males. Despite our best efforts, the pool of female esports athletes (i.e., professional players) is much smaller than their male counterparts, due to a significant under-representation in esports compared to casual gaming. Although our study is the first to present sleep data on female esports athletes, further efforts must be made to recruit and better understand the sleep of female esports athletes. Third, we cannot rule out a time-of-day influence on reaction-time testing, due to the testing time window of 9 h between teams. Although this window of time was a function of the busy schedules of teams, future research should aim to reduce the potential influence of time-of-day. Finally, the study was not pre-registered with the World Health Organization, which was an oversight due to the speed in which the study had to be deployed.

## 5. Conclusions

The present study was the first to trial a brief sleep intervention in an esports population and sets a benchmark to compare future interventions. Sleep knowledge increased, and there were modest short-term sleep benefits (subjective and objective), but this did not translate into improved mood (i.e., depression and anxiety) and cognitive performance (i.e., PVT). Further research is required to enhance the sleep, mood, and performance outcomes of sleep interventions in esports. More specifically, a stepped-care model that addresses the individual sleep needs of esports athletes (i.e., which provides an increased therapeutic dose of sleep knowledge and skills to those who need it) should be investigated next. Importantly, this type of intervention framework may not only be effective, but also feasible, by being able to fit within the constraints of esports athletes’ training and competition schedules.

## Figures and Tables

**Table 1:** Characteristics of study participants by country of origin.

Measure	South Korea *N* = 34	United States *N* = 7	Australia *N* = 15	Total Sample *N* = 56	F (*p*)
Age (years)	21.0 (2.42)	21.9 (3.16)	20.0 (1.94)	20.9 (2.43)	1.64 (0.20)
BMI (kg/m^2^)	24.2 (5.39)	25.0 (5.88)	22.4 (4.69)	23.8 (5.26)	0.82 (0.44)
Training hours per day	12.71 (2.04)	6.93 (1.79)	5.80 (2.10)	10.13 (3.81)	70.27 (0.001)
Career length in yrs	2.9 (1.6)	2.9 (1.5)	1.8 (1.2)	2.6 (1.6)	2.74 (0.074)
					Chi-square (*p*)
Female sex, *n* (%)	0 (0%)	2 (29%)	0 (0%)	2 (4%)	14.52 (0.001)
Sleep disturbance pre-competition, *n* (%)	14 (42%)	3 (43%)	10 (67%)	27 (49%)	2.55 (0.28)
Sleep medication use *n* (%)	0 (0%)	0 (0%)	1 (7%)	1 (2%)	2.78 (0.24)
					Kruskal-Wallis
Caffeine intake (mg per day), *n* (%)					3.94 (0.14)
<100	14 (44%)	4 (57%)	8 (67%)	26 (51%)	
100–200	7 (22%)	2 (29%)	4 (33%)	13 (25%)	
300–400	7 (22%)	1 (14%)	0 (0.0%)	8 (16%)	
>400	4 (12%)	0 (0%)	0 (0%)	4 (8%)	

Please note: To test for differences between countries of origin on age, BMI, training hours, and career length, we used ANOVA (F = Fisher–Snedecor). Least-significant difference post hoc testing was conducted to interpret significant effects. Chi-square was used to establish the frequency for sex and sleep disturbances pre-competition. Differences in caffeine intake were examined with Kruskal–Wallis test.

**Table 2:** Sleep diary estimates of sleep parameters pre- and post-intervention.

Sleep Parameter	Main Effect—Time		Pre-Intervention M(SE)	Post-Intervention M(SE)	*M*_diff_
	*t*	*p*			
TST (hrs)	1.67	0.09	7.4 (0.38)	7.5 (0.38)	0.1
SOL (mins)	−2.31	0.02 *	28.3 (9.12)	25.4 (9.12)	−2.9
WASO (mins)	−1.12	0.26	9.6 (8.67)	8.2 (8.68)	−1.4
SOT (hh:mm)	−2.28	0.03 *	03:42 (00:38)	03:30 (00:37)	−00:12
WUT (hh:mm)	−1.45	0.15	11:06 (00:30)	10:54 (00:33)	−00:12
SE (%)	2.88	0.004 *	91.5 (0.03)	92.6 (0.03)	1.1

Abbreviations: TST = total sleep time; SOL = sleep onset latency; WASO = wake after sleep onset; SOT = sleep onset time; WUT = wake-up time; SE = sleep efficiency. * indicates significant *p* value (*p* ≤ 0.05).

**Table 3:** Wrist activity monitor estimates of sleep parameters pre- and post-intervention.

Sleep Parameter	Main Effect—Time		Pre-Intervention M(SE)	Post-Intervention M(SE)	*M*_diff_
	*t*	*p*			
TST (hrs)	1.87	0.06	6.9 (0.31)	7.05 (0.32)	0.15
SOL (mins)	−0.77	0.44	31.6 (4.09)	30.3 (4.11)	−1.3
WASO (mins)	−1.13	0.26	44.7 (11.4)	42.5 (11.4)	−2.2
SOT (hh:mm)	−2.53	0.01 *	04:00 (00:26)	03:42 (00:26)	−00:18
WUT (hh:mm)	−1.17	0.24	11:36 (00:23)	11:24 (00:23)	−00:12
SE (%)	1.18	0.24	80.3 (2.84)	80.8 (2.84)	0.5

Abbreviations: TST = total sleep time; SOL = sleep onset latency; WASO = wake after sleep onset; SOT = sleep onset time; WUT = wake-up time; SE = sleep efficiency. * indicates significant *p* value (*p* ≤ 0.05).

**Table 4:** Sleep, mood, and PVT scores pre- and post-intervention.

Measure	Main Effect—Time		Pre-Intervention M (SE)	Post-Intervention M (SE)	Effect Size (95% CI)/*M*_diff_
	*F*	*p*			
ISI	12.79	0.001 *	10.50 (1.52)	8.47 (1.47)	0.47 (0.08, 0.84) **
PDSS	5.32	0.02 *	14.75 (1.81)	16.07 (1.82)	−0.23 (−0.61, 0.14)
STAI-Y	1.03	0.31	44.96 (3.30)	43.78 (3.39)	0.12 (−0.26, 0.49)
CES-D	0.01	0.94	27.33 (1.85)	27.40 (1.99)	−0.009 (−0.38, 0.36)
Mean reaction time (msecs)	0.26	0.61	251.3 (10.90)	249.6 (11.11)	−1.7
Lapses(≥500 ms)	2.04	0.16	3.0 (1.03)	2.5 (1.02)	0.2 (−0.18, 0.58)

Abbreviations: ISI = Insomnia Severity Index; PDSS = Pediatric Daytime Sleepiness Scale; STAI-Y = State-Trait Anxiety Inventory; CES-D = Centre for Epidemiological Studies Depression. Please note that Cohen’s d is used to express effect size for all variables apart from mean reaction time, which is expressed with *M*_diff_. * indicates significant *p* values. ** indicates meaningful Cohen’s d.

## Data Availability

The data presented in this study are available on request from the corresponding author. The data are not publicly available due to participants’ privacy protection.

## Supplementary Materials

The following are available online at https://www.mdpi.com/article/10.3390/ijerph19074146/s1, File S1: Sleep Quiz.
