# Supplementary material for: Evaluation of a Brief Sleep Intervention Designed to Improve the Sleep, Mood, and Cognitive Performance of Esports Athletes
Source: Int J Environ Res Public Health. 2022 Mar 31;19(7):4146. doi: 10.3390/ijerph19074146 (PMC8998799; doi:10.3390/ijerph19074146)
Supplement: Supplementary file 1 [file ijerph-19-04146-s001.zip › ijerph-1612745-SI.pdf]

Sleep Quiz

Please read through each question and circle the correct answer.

1. **Sleep is a time when your body and brain shut down for rest and relaxation.**

   True False Don’t Know

2. **Most young adults (18-25) need between 7-9hrs of sleep each night.**

   True False Don’t Know

3. **Getting one hour less sleep per night than I need will not have any effect on Esports performance.**

   True False Don’t Know

4. **Taking up to 30mins to get to sleep is normal.**

   True False Don’t Know

5. **Some technology use at night is ok as long as I try to fall asleep around the same time each night.**

   True False Don’t Know

6. **Caffeine should be used as the primary strategy to manage sleep loss and sleepiness in Esports.**

   True False Don’t Know

7. **6hrs of sleep or less per night over a few weeks has the equivalent effect on performance as going 48 hrs without sleep.**

   True False Don’t Know

8. **Deep sleep is the best type of sleep related to learning and memory.**

   True False Don’t Know

9. **Unlike traditional sports, Esports has very few risk factors that can lead to poor sleep.**

   True False Don’t Know

10. **One process that controls when I sleep and wake-up is called the body clock.**

    True False Don’t Know

11. An Eathlete will always know if their performance is being affected by sleep loss.

True | False | Don't Know

12. Tactical awareness (i.e. executing a game plan, making effective decisions, reacting to changes in game plans) is unaffected by sleep loss.

True | False | Don't Know

13. I should prioritise healthy sleep habits but I shouldn't stress about my sleep.

True | False | Don't Know

14. Getting more sleep after being sleep deprived will improve my reaction time, mood and reduce fatigue.

True | False | Don't Know

15. One consequence of sleep loss on performance is persevering with a strategy even though it is not working.

True | False | Don't Know

16. Waking up during the night is abnormal.

True | False | Don't Know

Answers

1. False
2. True
3. False
4. True
5. True
6. False
7. True
8. False
9. False
10. True
11. False
12. False
13. True
14. True
15. True
16. False
